# Targeting Multiple End Organs in Lupus and Other Systemic Rheumatic Diseases by Inhibiting Bruton’s Tyrosine Kinase

**DOI:** 10.3389/fimmu.2022.893899

**Published:** 2022-07-08

**Authors:** Yong Du, Ling Lei, Huihua Ding, Yanping Chen, Simanta Pathak, John Hicks, Phuongthy T. Tran, Minghua Wu, Betty Chang, Uwe Wirtz, Chandra Mohan

**Affiliations:** ^1^ The Department of Biomedical Engineering, University of Houston, Houston, TX, United States; ^2^ Department of Pathology, Texas Children’s Hospital, Houston, TX, United States; ^3^ Division of Rheumatology and Clinical Immunogenetics, University of Texas Health Science Center at Houston, Houston, TX, United States; ^4^ Summit Therapeutics, Menlo Park, CA, United States; ^5^ AbbVie, Inc., Precision Medicine, San Francisco, CA, United States

**Keywords:** lupus nephritis, B-cell, signaling, Bruton tyrosine kinase, scleroderma

## Abstract

Bruton tyrosine kinase (Btk) plays a vital role in activating and differentiating B-cells and regulating signaling in myeloid cells. Indeed, the potential use of Btk inhibitors in preventing lupus has been reported. Here, we extend these observations to 4 additional models of end-organ inflammation: (a) BWF1 lupus nephritis mice, (b) anti-GBM nephritis, (c) bleomycin-induced systemic sclerosis like skin disease, and (d) bleomycin-induced lung disease. In agreement with the previous studies, BTK inhibitor (BTKB66) treatment was effective in treating lupus nephritis in terms of reducing renal damage both functionally and histologically, accompanied by significant decrease in proteinuria. Both low-dose and high-dose BTKB66 profoundly blocked renal disease in the anti-GBM nephritis model, with efficacy that was comparable to that seen with dexamethasone. This study provides the first evidence that BTK inhibition has both therapeutic and preventative effects in bleomycin-induced SSc-like disease, in terms of reducing skin thickness, fibrosis, collagen deposition, and inflammation. Likewise, significantly lower lung inflammatory cell infiltration was observed after treatment with BTKB66. Therapeutic benefit was associated with lower numbers of macrophages, proliferating macrophages and activated T-cells in the respective injured organs. The observation that these immune cells play key roles in driving end organ inflammation in multiple systemic rheumatic diseases have broad implications for the use of BTKB66 in managing patients with systemic rheumatic diseases where multiple end organs are afflicted, including lupus and systemic sclerosis.

## Introduction

Bruton tyrosine kinase (Btk) is cytoplasmic nonreceptor protein tyrosine kinase in the Tec family. It is predominantly expressed in B cells, but also expressed in other hematopoietic cells including myeloid cells (1, 2). In the B-cell receptor (BCR) signaling pathway, the Src family kinase, spleen tyrosine kinase (SYK) initiates the activation of Btk, which enhances the catalytic activity of Btk and results in the activation of downstream NF-κB and MAP kinase pathways (1). Btk also participates in other signaling pathways including chemokine receptor signaling, FcγR mediated signaling, and toll-like receptor (TLR) signaling. Btk mutations in humans cause X-linked agammaglobulinaemia (XLA) which is characterized by a lack of peripheral blood B cells and immunoglobulins with intact T-cell function and cellular immunity (3, 4). Although the clinical use of Btk inhibitors has been limited to B-cell malignancies, animal studies have demonstrated the potential efficacy of Btk inhibition in autoimmune diseases, particularly rheumatoid arthritis and SLE (5–10). In this study, we examine if Btk inhibition might be suitable for ameliorating disease in additional target organs commonly afflicted in systemic rheumatic diseases.

Systemic sclerosis (SSc), another systemic autoimmune disease, is marked by sclerotic and fibrotic changes in multiple organ systems, including the skin, pulmonary and gastrointestinal systems. B cells have been shown to play pathogenic roles in this disease. Several genome-wide association (GWS) studies have identified B cell related genes in SSc, such as B-cell scaffold protein with Ankryn repeats (BANK1), B lymphoid kinase (Blk), and Tumor Necrosis Factor Superfamily-4 (TNFSF4) (11–13). In animal models, the link between B-cell hyperactivity and fibrosis has been established in the tight skin mouse (TSK) model of systemic sclerosis (14). A deficiency of the CD19 gene leads to diminished skin thickening and lung fibrosis in the Bleomycin (BLM)-induced SSc mouse model when compared to BLM-treated WT littermates, indicating that B cells are actively involved in the fibrotic process that mediates skin and lung disease (15). Moreover, BAFF blockade and B-cell depletion therapies have also been effective in some patients with this disease (16–18). However, long-term clinical outcome in this disease is still dismal, particularly due to progressive pulmonary fibrotic disease (19). Considering the significant role of B cells (and myeloid cells) in the development of SSc, we examined the effect of BTK inhibition on both skin and lung fibrosis in a BLM-induced mouse model of this disease, as well as in two additional mouse models of end organ inflammation, as described below.

Systemic lupus erythematosus (SLE) is a systemic autoimmune disease that affects multiple organs and produces a wide array of autoantibodies. The kidney is one of the most commonly targeted organs, and lupus nephritis (LN) contributes significantly to morbidity and mortality in SLE (20). It is associated with an estimated 6-fold increase in mortality compared to the general population (21). Partly due to the complexity and heterogeneity of human SLE, murine models have been used to investigate the pathogenesis and therapeutic targets in LN. Abnormal B-cell activation and immune complex formation are major immunopathological features of LN (22). NZB mice B cells have demonstrated a higher level of phosphorylation of tyrosine residues and a strong response to BCR mediated activation (22, 23). In NZBW F1 mice, the activation of FcRs on circulating hemopoietic cells is required for immune-complex mediated disease pathogenesis (24). Since Btk participates in both BCR and FcγR signaling pathways, it is considered an attractive target in LN treatment. Treating NZBW/F1 lupus mice with the Btk inhibitor RN486 ameliorated lupus disease progression as determined by histologic and functional analyses of glomerulonephritis (9). Similarly, in MRL/lpr mice, another spontaneous lupus murine model, Btk inhibition was able to normalize proteinuria with early treatment, and reverse established proteinuria when administrated at the late phase of the disease (25). Similar findings have been reported in C57BL/6-based congenic models of lupus (8).

Anti-Glomerular Membrane Nephritis (anti-GBM) is a classic antibody-mediated autoimmune disease, characterized by marked crescent formation and progressive renal failure. Cumulative evidence has implicated that both cell-mediated processes and humoral mechanisms in the development of renal injury in anti-GBM disease. For example, transferring heterologous antibodies from humans to new world monkeys could induce proteinuria and renal injury, while plasmapheresis is the standard treatment for human anti-GBM disease (26). Rituximab, a B-cell depleting agent, has also been effectively used for the treatment of anti-GBM disease (27). Given the demonstrated role of B-cells and myeloid cells in this disease, we reasoned that Btk inhibition would also be therapeutically effective in this disease.

## Material and methods

### Animal Experiment

All mice used in these studies were purchased from The Jackson Laboratory (Bar Harbor, ME). C3H/HeJ strain was used to generate the Bleomycin-induced systemic sclerosis (SSc) murine model, 129x1/svJ mice were used for anti-GBM nephritis, and NZB/WF1 is a typical spontaneous lupus nephritis (SLN) model. C57BL/6J mice were used as controls and for the *in vitro* experiments. All mice were housed in a pathogen-free animal facility at the University of Houston, TX, USA. All experiments were conducted in accordance with the Guide for the Care and Use of Laboratory Animals (NAP 2011) and have been approved by the Institutional Animal Care and Use Committee at the University of Houston.

### BTK Inhibitor (BTKB66)

BTKB66 (Pharmacyclics Inc.) was formulated in 0.16M citric acid to a final concentration of 4 mg/ml (28). The drug was administrated by oral gavage at 25 mg/kg or 50 mg/kg per day for 4-8 weeks, as indicated in each experiment. The control group was given 0.16M citric acid using the same schedule. The dose and route of administration was chosen based on previously established concentrations needed for significant inhibition of Btk activity using this drug (28) or its structural analogue ibrutinib (8). The chosen drug, BTKB66, has been shown to be selective for BTK as it targets BTK with an IC50 of 13.3 nM, targeting TEC, BLK, FGR and PTK6 with IC50 values of 195-493 nM, but with absence of targeting of 100 other kinases at IC50 values >1000, including Itk (28). The safety profiles of these inhibitors have previously been reported (29). The salubrious effect of BTKB66 on liver damage has been examined and reported in detail (28).

### BLM-Induced SSc Mouse Model and BTKB66 Treatment

A murine model that mirrors the inflammation-driven aspects of human SSc was induced by intradermal injection of Bleomycin 100 μg per day over a total of 4 weeks in C3H/HeJ mice. To gauge the preventative and therapeutic effects of BTK inhibition on SSc-like disease, mice were randomly categorized into two groups: 1) BTKB66-4W: the BTK treatment was initiated at the same time as the BLM injection; 2) BTKB66-8W: BTK inhibitor treatment was started 4 weeks after BLM injection, when SSc disease was already present. In both groups, BTKB66 and the vehicle control were administered by oral gavage once per day at the dosage of 25 mg/kg/d, over 4 weeks. At the end of treatment, skin and lung tissue were collected for pathology evaluation, skin thickness measurement, and Collagen IV content determination. The skin and lung collagen content were determined using commercial kits, as detailed in **Supplementary Information**. The treatment was repeated twice, with a total of 10-15 mice per group.

### Spontaneous Lupus Nephritis and BTKB66 Treatment

26-week old female NZB/W F1 mice were randomized into treatment and control groups (n=10 in each group). BTKB66 was administered by oral gavage once per day at the dosage of 25 mg/kg/d, while the control group was given the vehicle buffer. Blood and urine samples were collected once per month for renal function tests and autoimmunity detection. One kidney from each mouse was used for pathology evaluation, and the other was used for flow cytometry analysis.

### Anti-GBM Nephritis Model and BTKB66 Treatment

Thirty-two 129x1/svJ mice were used to induce anti-GBM nephritis, as described (30). In brief, mice were pre-immunized using Rabbit IgG (250μg/20g mouse body weight, Sigma) mixed with CFA (100 μL/20g mouse body weight, Sigma), then, i.v injected with 200 µl rabbit anti-mouse GBM serum on D5. The mice were categorized into BTKB66 low dose (25 mg/kg/d), BTKB66 high dose (50 mg/kg/d), Dexamethasone (Dex, 0.4 mg/kg/d), or the PBS control group (n=6-8, per group). The treatment was started three days after anti-GBM injection. Blood and 24hr urine samples were collected once per week for BUN and 24-hrs proteinuria measurements. Kidneys were subjected to pathology assessment.

### The Impact of BTK Inhibitor on B Cells and Bone Marrow-Driven Macrophages

B cells were purified from 2-month-old C57BL/6J mice spleens by negative selection using a commercial bead kit purchased from Stem Cells. Bone marrow- derived macrophages (BMDM) were generated from B6 bone marrow cells incubated with 10 ng/ml MCSF for five days. B cells were stimulated with anti-IgM or anti-IgM with different concentrations of BTKB66, while BMDMs were incubated with 100 ng/ml LPS, or LPS with different concentrations of BTK inhibitor (10, 100, 1000 ng/ml) for 24hrs. Activation of B cells and BMDMs were accessed by flow cytometry using the indicated cell surface markers. Pro-inflammatory cytokine levels in the BMDM culture medium were measured using commercial ELISA kits (R&D, USA)

### Pathology Evaluation

At the end of each experiment, the targeted tissues were harvested for pathology evaluation. For SSc mice, the skin and lung were collected for hematoxylin and eosin (H&E) and Masson Trichome staining, while kidneys from the SLN and anti-GBM nephritic mice were subjected to H&E and periodic-acid Schiff (PAS) staining. For skin tissue, dermal thickness, defined as the thickness of the skin from the top of the granular layer to the junction between the dermis and subcutaneous fat, was measured. A blinded pathologist (JH) read and assessed all histology changes blindly using standard scoring systems. For dermatologic changes, epidermal and dermal inflammation, skin fibrosis, and vasculitis were evaluated. For lung histopathology grading, the severity of inflammatory infiltration, airspace dilation, interstitial fibrosis, and vasculitis were determined. Renal pathology changes were scored using a well-established grading method, which evaluated the histological changes in glomerular and tubulointerstitial lesions, and the severity of crescent formation, as described (30, 31). The scoring criteria are detailed in **Supplementary Information**.

### Flow Cytometry Analysis of Renal Infiltrating Inflammatory Cells

To analyze renal inflammation, renal infiltrating inflammatory cells were extracted and analyzed using flow cytometry. In brief, one kidney from each SLN mouse underwent flow cytometry analysis. Kidneys were minced with a razor blade and digested with 1mg/ml collagenase type 4 (Cat.# LS004188, Worthington Biochemical Corp, NJ, USA) for 30 minutes at 37°C in a shaking incubator. After incubation, cells were immediately moved onto ice, and the digestion was stopped by adding RPMI media 1640 (Cat. # 12633, Thermo Fisher Scientific Inc, MA, USA). Red blood cells were lysed using RBC lysing buffer (Sigma). Cell clumps were disrupted using a syringe with 22G needle and then forced through a 70 µm filter. For flow cytometric analysis, cells were stained with antibodies against B220, CD3, CD4, CD8, CD11b, CD11c, CD21, CD23, CD25, CD45, CD69, CD80, CD86, F4/80, Gr1 (BD Biosciences, CA, USA). Approximately 50,000 events were acquired from each sample. All samples were run on a NovoCyte flow cytometer (ACEA Biosciences, CA, USA). Analysis was performed using Novoexpress (ACEA Biosciences, CA, USA). Experimental details are included in **Supplementary Information**.

### Tissue Macrophage Staining

Kidneys from anti-GBM nephritis mice, and skin and lung tissue from mice with SSc-like disease were subjected to immunofluorescence staining for macrophages. Anti-Iba1 (Wako, Cat.# 019-19741, 1:300) was used to label macrophages, while anti-PCNA (Abcam, Cat.#ab29, 1:200) was used to stain proliferating cells. Briefly, after deparaffinization and rehydration, sections were boiled for antigen-retrieval using Citrate buffer (PH6.0). Then, sections were blocked with 5% goat serum with 0.25% Triton X-100 for 2hrs and incubated with primary Abs overnight at 4°C. Alexa Fluor 488, Goat anti-Rabbit IgG (H+L) and Alexa Fluor 555, Goat anti-mouse IgG (H+L) were added as 2^nd^ Abs (1;1000) for 2hr at RT. After final washing and mounting, a Nikon confocal microscope was used for imaging. For each section, 10 random fields were selected to count positive staining. Iba1^+^ PCNA^+^ cells are indicative of proliferating macrophages.

### Statistical Analysis

All data are expressed as mean ± SD. Comparison between two groups was performed using a parametric Student t-test or non-parametric Mann Whitney U test (when data was not normally distributed), as indicated in the figure legends. Comparison between multiple groups was conducted using one-way ANOVA followed with Tukey’s *post-hoc* test. *P* values < 0.05 were considered statistically significant. All statistical analysis was performed using GraphPad Prism (V.10.0, GraphPad, CA, USA).

## Results

### BTK Inhibition Yields Strong Therapeutic Efficacy Against BLM-Induced SSc-Like Fibrotic Skin Disease

**Figure 1:**
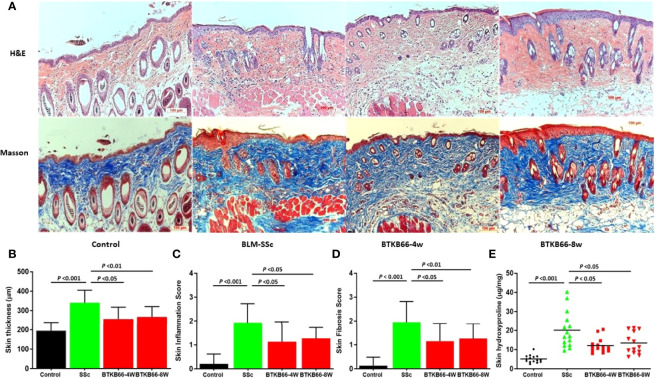
BTK inhibition ameliorates skin disease in BLM-SSc mice, with reduced skin thickness, inflammation, fibrosis score and hydroxyproline content. **(A)** Representative H&E (top) and Masson (bottom) staining from each group are shown (n= 10-15 per group, 20x). Compared to normal skin, BLM-SSc skin exhibited a significant increase in dermal thickness, a decrease in the amount of subcutaneous fat tissue, an infiltration of inflammatory cells, and robust deposition of collagen in the skin. **(B)** both the BTKB66-4W and BTKB66-8w treated groups exhibited lower skin thickness **(B)**, decreased inflammatory cell infiltration **(C)**, lower skin fibrosis score **(D)**, and less skin hydroxyproline content **(E)**. The treatment was repeated twice, with a total of 10-15 mice per group, one-way ANOVA, *P <*0.05.

Compared to normal skin, BLM-SSc skin exhibited a significant increase in dermal thickness, prominent inflammatory cell infiltration, robust deposition of collagen in the skin, and less subcutaneous fat tissue, as shown in **Figure 1A**. However, both the BTK-4W and BTK-8w treated groups exhibited less skin fibrosis, less collagen deposition, and decreased inflammatory cell infiltration. Pathology assessment indicated that both BTK-treated groups had significant decrease in skin thickness (**Figure 1B**), less inflammatory cell infiltration (**Figure 1C**), and a lower skin fibrosis score (**Figure 1D**). Lower hydroxyproline content was noted after BTK treatment, compared to that of the PBS control group (**Figure 1E**, 2-tailed t -test, *p <*0.05, n= 10-15 each group).

### BTK Inhibition Ameliorates Lung Inflammation in BLM-Induced SSc-Like Disease

**Figure 2:**
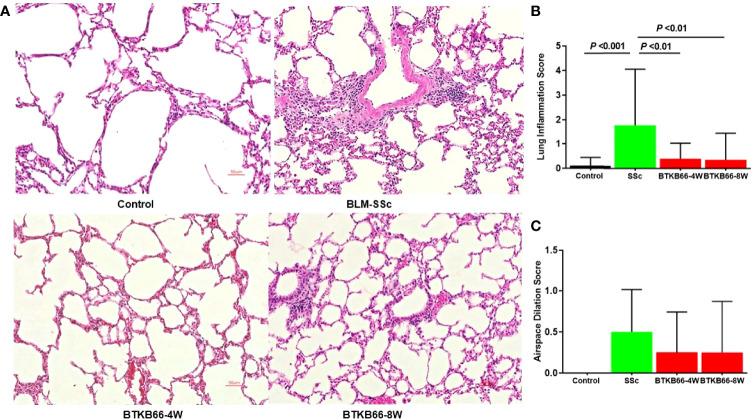
The effect of BTK Inhibition on lung inflammation in BLM-SSc mice. Pulmonary histopathologic changes, including inflammation, airspace dilation, fibrosis, and vasculitis, were evaluated in BLM-SSc mice. **(A)** Representative H&E-stained lung tissue images from each group are shown (n= 10-15, 20x). **(B)** Displayed are inflammation score and airspace dilation score in each group (n= 10-15; one-way ANOVA). SSc mice exhibited higher lung inflammation score, compared to the BTK-treated group. **(C)** Increased airspace dilation score was assessed in all SSc mice. However, no significant difference was detected.

As lung fibrosis is a major cause of death in SSc patients, we also investigated the effect of the BTK inhibitor on lung histopathologic changes, including inflammation, airspace dilation, fibrosis, and vasculitis. As shown in **Figure 2**, BTK-treated mice exhibited significantly lower inflammation with fewer inflammatory cell infiltrates compared to BLM-SSc lungs. Since lung fibrosis was not prominent in BLM-SSc mice, we were not able to assess the impact of BTK inhibition on this phenotype. No significant hydroxyproline content differences were noted in the lungs of these mice (data not shown).

### BTK Inhibition Improved Renal Damage in SLN Model and in Anti-GBM Model

Several studies have examined the effect of BTK inhibition on SLN mice and found that BTK inhibition ameliorates renal, skin, and brain disease in MRL/lpr mice (7–10). Taking advantage of the improved selectivity and specificity of BTKB66 used in this study, we extended our studies to another commonly studied SLN murine model: NZB/WF1. Treatment was started at 26 weeks of age, at which point some mice were proteinuric. After 8 weeks of treatment, BTKB66 was associated with less renal damage and renal dysfunction in NZB/WF1 mice as evidenced by decreased 24-hr-proteinuria (**Supplementary Figure 1A**). Consistent with improved renal function, BTKB66 treated mice exhibited significantly lower GN scores compared to the control group (1.0 ± 0.38 *vs* 2.7 ± 0.57, *P* =0.02, **Supplementary Figures 1C, E**).

To explore the effect of BTKB66 on systemic autoimmunity, serum auto-Ab levels were measured by ELISA and the phenotype of splenocytes was determined by flow cytometry. At the end of treatment, the average spleen to body weight ratio was 0.0024 ± 0.0003 in the treatment group and 0.0046 ± 0.0013 in the control group (*p* = 0.03), indicating that BTKB66 treatment was associated with ~41% reduction in spleen:body weight ratios compared to the control group (**Supplementary Figure 2A**). Serum total IgG, IgM, and anti-dsDNA levels were compared between the treatment and control groups. Total IgM and IgG levels were similar between the treatment and control groups (**Supplementary Figures 2C, D**). Similarly, we did not find significant differences in autoantibody levels between the two groups. However, there was a trend towards lower IgG anti-dsDNA autoantibody levels in the treatment group (**Supplementary Figures 2E, F**)

Anti-GBM nephritis shares similar cellular and molecular downstream mechanisms with lupus nephritis (22), although it results in nephritis that is more acute in nature, and less dependent on systemic autoimmunity. Hence, we also examined the therapeutic efficacy of BTK inhibition on anti-GBM nephritis. As shown in **Supplementary Figure 3**, the anti-GBM challenged mice exhibited worse renal function, with significantly higher 24hr-proteinuria (A), increased BUN levels (B), and higher GN/TIN scores (C-E), compared to the BTKB66-treated mice. The BTKB66 low and high dose treatment groups both demonstrated therapeutic efficacy with significantly lower levels of BUN and 24-hr proteinuria levels, with significantly improved GN and TI disease scores, comparable to the therapeutic efficacy seen with dexamethasone treatment.

### BTK Inhibition Was Associated With Reduced Systemic and End-Organ T Cell and Myeloid Cell Infiltration and Activation

To investigate the underlying pathogenic mechanism mediated by BTKB66 treatment, we next examined the cell populations in both the kidneys (**Supplementary Table S1**) and the spleen (**Supplementary Table S2**) of NZBW F1 mice after 8-week BTK inhibition, using flow cytometry.

Activated CD3^+^ T cell and CD4^+^ T cell numbers in spleen was lower by 36% and 37% respectively in the BTKB66 treated group as compared to the control group (both, *p* = 0.03, **Supplementary Figures 4A, C**). Total CD4^+^ T cell numbers decreased by 13% and total CD8^+^ T cell numbers increased by 37% in the spleen (*p*= 0.01 and *p* = 0.04, respectively, **Supplementary Figure S4B**). However, we did not detect any difference in B cell numbers or subsets, including total B cells, follicular B cells, and marginal zone B cells, following BTKB66 treatment.

In addition, BTKB66 had a strong inhibitory effect on the numbers of intra-renal activated T-cells and myeloid cells, as detailed in **Supplementary Table S1**. Mice in BTKB66 treated group exhibited reduced total CD4^+^ and activated CD4^+^ T cells and reduced CD11b^+^, and CD86^+^ cells. There was no significant difference in the total number of CD45^+^ Gr-1^+^ cells between these two groups. However, CD45^+^Gr1^+^ cells in the treatment group expressed less CD86 than the controls(4.06 x 10^3^ *vs* 1.52 x 10^5^, respectively, *P <*0.01) indicating decreased activation. Additionally, F4/80^+^ and activated F4/80^+^ macrophages were lower in the treatment group (4.60 x 10^3^ *vs* 1.07 x 10^4^ respectively, *P <*0.01)

Collectively, these results indicate that the reduced renal pathology; proteinuria; and splenomegaly in lupus mice following BTK inhibition is associated with significantly lower peripheral and intra-renal T-cell numbers, and myeloid cell activation, although the impact on B-cells and autoantibodies was minimal.

### BTK Inhibition Is Associated With Reduced Both B Cell and Macrophage Activation *In Vitro*

**Figure 3:**
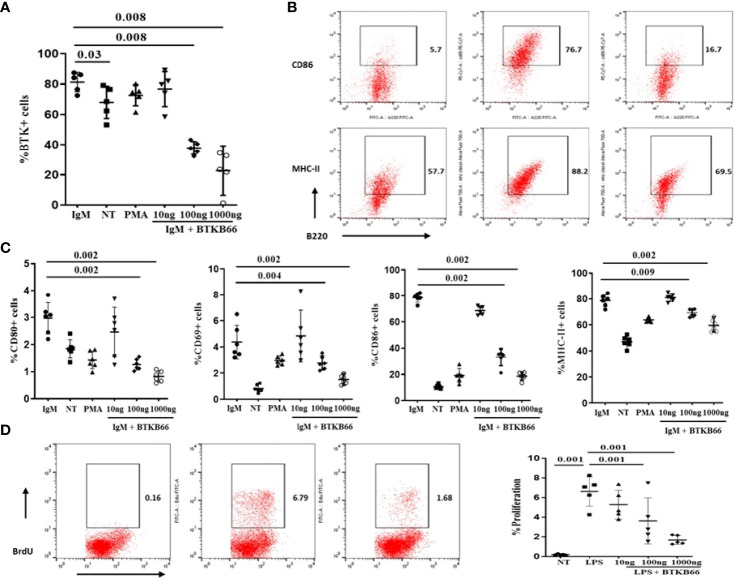
BTK inhibition dampened B cell activation in a dose-dependent manner *in vitro*. B cells were isolated by negative selection using commercial bead-based kits. Then these purified B cells were seeded at 1x 10^6^ cell per well and incubated with either anti-IgM or LPS, PMA, with or without BTKB66 at different concentrations (10, 100, 1000 ng/ml) for 24hrs (n =6 per group). Cells without any stimulation served as control (NT). **(A)** BTK inhibition reduced BTK phosphorylation in B cells. **(B)** Representative flow cytometry images of B cell activation showed decreased CD86^+^B220^+^; MHC-II^+^ B220^+^ cells after coculture with BTKB66. **(C)** From the left to right: CD80^+^ B220^+^; CD69^+^B220^+^, CD86^+^B220^+^; MHC-II^+^ B220^+^. All 4 of these markers represent different B-cell activation markers. The BTK inhibitor demonstrated a dose-dependent inhibition of B cell activation. **(D)** Inhibitory effect BTKB66 on LPS-stimulated B cell activation. One-way ANOVA, followed with Turkey test was used to compare the difference between two groups.

Both B cells and macrophages play critical roles in the development of various autoimmune diseases including SSc and lupus nephritis, and are both impacted by BTK activation (6, 10, 16). The immunomodulatory effects of BTK inhibition on B cells and macrophages was explored following *in vitro* culture. B cells were purified from 2-month-old C57BL/6J mice spleen by negative selection. One group of B cells was stimulated with anti-IgM or anti-IgM with different concentrations of BTKB66 (10,100, and1000 ng/ml) for 24hrs. As shown in **Figure 3**, BTK inhibition reduced BTK phosphorylation in B cells (**Figure 3A**). The percentages of CD86^+^B220^+^, CD80^+^B220, MHC II^+^B220^+^, and CD69^+^B220^+^ cells were significantly reduced after co-culture with BTKB66, in a dose-dependent manner (**Figures 3B, C**). Another group of B cells was stimulated with LPS, with or without BTKB66. As noted above, a dose-dependent suppressive effect of BTKB66 on LPS-induced B-cell proliferation was observed (**Figure 3D**).

**Figure 4:**
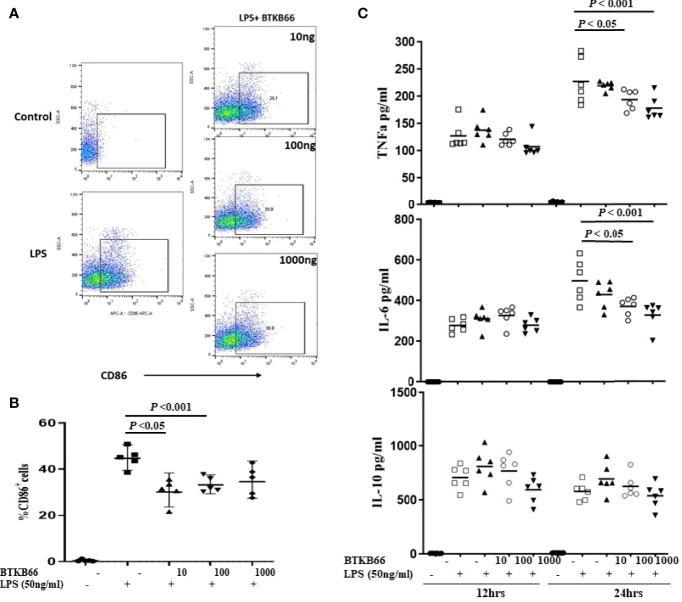
BTK inhibition reduced LPS-induced BMDM activation and pro-inflammatory cytokine production *in vitro*. Bone marrow derived macrophages (BMDMs) were generated from C57BL/6J mouse bone marrow with MCSF (10 ng/ml) incubation for 5 days. Then, these BMDM cells were incubated with LPS (50 ng/ml), or LPS with varying doses of BTKB66 (10, 100, 1000 ng/ml) for 24 hrs (n = 6 per group). Cells were harvested at 24 hrs for flow cytometry, and culture supernatant was collected at 12 hrs and 24 hrs for pro-inflammatory cytokine detection. BMDMs without any stimulation served as control (NT). Shown are **(A)** Representative flow cytometry images of Macrophage activation showed decreased CD86^+^ macrophage after coculture with BTKB66. **(B)** Percentage of CD86^+^ macrophages, **(C)** TNF-α, IL-6, IL-10 levels in cell culture supernatant. The levels of pro-inflammatory cytokines, TNF-α and IL-6, were reduced by BTK inhibition in a dose dependent manner. No changes in IL-10 could be detected (one-way ANOVA).

BMDMs were generated from C57BL/6J mouse bone marrow cells with 5-days MCSF (10 ng/ml) incubation and then stimulated with LPS (50ng/ml), or LPS with different concentrations of BTKB66 (n = 6 per group). Culture supernatant was collected at 12hrs and 24hrs for pro-inflammatory cytokine detection, and cells were harvested at 24 hrs for FACS. As **Figure 4** indicates, the percentage of CD68^+^ BMDMs was increased after 24 hrs LPS stimulation, but it was significantly reduced in all BTK inhibition groups. Similarly, TNF-α and IL-6 levels in cell culture supernatant were reduced following BTK inhibition in a dose dependent manner. However, we did not detect any difference in IL-10 levels among the groups. Together, this data supports the *in vitro* inhibitory effect of BTK inhibition on both B-cell and macrophage activation, although the *in vivo* changes in B-cells were not detected.

### BTK Inhibition Significantly Inhibited *In Vivo* Macrophage Infiltration and Activation

**Figure 5:**
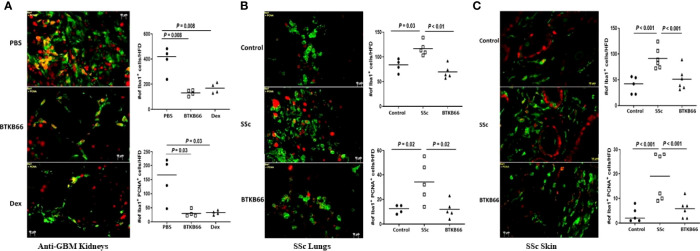
BTK inhibition was associated with significantly lower macrophages infiltration into targeted end-organs. Renal tissue from anti-GBM nephritis model, and skin and lung tissue from BLM-SSc mice were interrogated for macrophages (Iba1^+^, green color) and proliferating cells (PNCA, red color). Ten random fields per section were selected for counting positive-stained cells. Shown are **(A)** representative image from PBS, BTKB66 and Dex groups among the anti-GBM nephritis mice; **(B)** representative lung macrophage infiltration image from Control, SSc and BTKB66 groups; **(C)** representative skin macrophage infiltration image from Control, SSc and BTKB66 groups. For each tissue, total macrophage counts, and proliferating macrophage counts are plotted. In all tissues, BTKB66 was associated with significantly reduced numbers of macrophages and proliferating macrophages (n= 4-6 per group, one-way ANOVA).

As **Figure 5** (left) shows, Iba1^+^ positive cells and Iba1^+^ PCNA ^+^ cells were increased in the kidneys of anti-GBM nephritic mice treated with PBS (PBS group), while it was significantly lower in BTKB66 treated mice, comparable to the effects of Dex. Similarly, both skin (**Figure 5**, right) and lung (**Figure 5**, middle) tissue from the SSc group exhibited significantly increased infiltration by macrophages and proliferating cells (including proliferating macrophages), compared to that of the control group. With BTKB66 treatment, these cells were significantly lower, both in skin and lung. Collectively, these results suggest that BTK inhibition may significantly reduce target organ infiltration by macrophages, including proliferating macrophages.

## Discussion

Bruton tyrosine kinase (Btk) plays a vital role in activating and differentiating B-cells and regulating signaling in myeloid cells. Besides B-cell receptor signaling, Btk functions through multiple signaling pathways including CD40 ligation, Toll-like receptor triggering, and Fc-receptor signaling. Hence, it is likely that targeting Btk could prove beneficial in autoimmune diseases characterized by pathologic autoantibodies, macrophage activation, and myeloid-derived type I interferon responses (1, 32, 33). Given that these pathogenic mechanisms mediate multiple end organ diseases in systemic rheumatic diseases, it becomes important to systematically assess the impact of BTKB66 on various end organ diseases.

Indeed, the potential use of Btk inhibitors in treating lupus has been reported and reproduced since 2010 (7–10). Data from these studies suggested that BTK inhibitors may simultaneously target autoantibody-producing and effector cells in SLE, thus preventing renal injury and improving renal pathology. In the current study, we explored the efficacy of a BTKB66 on mice with spontaneous LN at the nephritic phase. In agreement with the previous studies, BTKB66 was effective in treating LN in terms of reducing renal damage both functionally and histologically, accompanied by significant decrease in proteinuria. The studies with the acute anti-GBM model further bolsters the therapeutic potential of BTKB66 in immune-mediated renal diseases. Indeed, both low-dose and high-dose BTKB66 profoundly blocked renal disease in this induced model, with efficacy that was comparable to that seen with dexamethasone.

However, unlike the previously reported effects of other Btk inhibitors on autoantibody production, this study using NZBW/F1 mice did not demonstrate significant inhibition of total immunoglobulins and autoantibodies, though a decreasing trend in IgG anti-dsDNA levels were noted following BTKB66 treatment. In contrast, we observed a significant shift in the T-cell population. BTKB66 treatment was associated with significantly reduced T-cell activation as indicated by the lower numbers activated total T cells and CD4+ T cells, both in the spleen and the kidneys. Similarly, our previous work using a BTKB66 in B6.*Sle1.Sle3* mice also showed dampened activation of T cells (8). Previously used Btk inhibitors have been shown to be irreversible interleukin-2-inducible kinase (ITK) inhibitors which drive Th1-based immune response in T cells (34). Since the BTKB66 used in this study does not inhibit T-cell kinases (28), and BTK is not expressed in T-cells, the observation of BTKB66’s effect on T cells clearly warrants further investigation.

Similar to SLE, B cells and myeloid cells play prominent roles in SSc pathogenesis. It is not surprising that deficiency of CD19 was protective against BLM-SSc disease and blocking B cell function reduced skin fibrosis in BLM induced SSc (16–19). Macrophages and plasmacytoid dendritic cells (pDCs) also have been shown to play a critical role in SSc. For example, pDCs from SSc patients highly express CXCL4 protein, which facilitates the release of IFN-γ, activates endothelial cells, and potentially promotes fibrosis (35). This study provides the first evidence that BTK inhibition has both therapeutic and preventative effects on a BLM-induced SSc model, in terms of reducing skin thickness, fibrosis, collagen deposition, and inflammation. Likewise, we observed significantly lower lung inflammatory cell infiltration after BTKB66 treatment, and a trend towards improved airspace dilation. Both skin and lung fibrosis are leading pathological changes in SSc, and lung involvement is the leading cause of mortality in this disease. These results raise hope that targeting BTK could have therapeutic potential in SSc, and this clearly merits further study.

Using *in vitro* cell culture, the completed studies reveal that BTKB66 could inhibit B-cell activation following IgM stimulation and B-cell proliferation following LPS stimulation in a dose-dependent manner, consistent with previous reports examining the role of BTK in B-cell signaling. Likewise, BTKB66 also inhibited BMDM activation and pro-inflammatory cytokine production *in vitro*. Given these *in vitro* findings, it is likely that several of the observed outcomes in the *in vivo* disease models reported here are mediated by direct targeting of these immune cells. The fact that these immune cells play key roles in driving end organ inflammation in multiple systemic rheumatic diseases, and previous reports of BTK inhibition of lupus and rheumatoid arthritis (5–10), taken together, have broad implications for the use of BTKB66 in managing patients with systemic rheumatic diseases where multiple end organs are afflicted.

## Data Availability Statement

The raw data supporting the conclusions of this article will be made available by the authors, without undue reservation.

## Ethics Statement

The animal study was reviewed and approved by IACUC, University of Houston.

## Author Contributions

YD, LL, HD, YC, SP, PT, and MW performed the experiments and collected data. YD, HD, and PT prepared the manuscript. YD, HD, and MW performed statistical analysis and data interpretation. JH conducted pathology evaluation. BC, UW, and CM oversaw the whole project, integrated data, and final approval of the manuscript and critical revision. All authors contributed to the article and approved the submitted version.

## Conflict of Interest

Author BC is employed by Summit Therapeutics, and author UW is employed by AbbVie/PCYC Translational Oncology.

The remaining authors declare that the research was conducted in the absence of any commercial or financial relationships that could be construed as a potential conflict of interest.

## Publisher’s Note

All claims expressed in this article are solely those of the authors and do not necessarily represent those of their affiliated organizations, or those of the publisher, the editors and the reviewers. Any product that may be evaluated in this article, or claim that may be made by its manufacturer, is not guaranteed or endorsed by the publisher.
