# Supplementary material for: Targeting Multiple End Organs in Lupus and Other Systemic Rheumatic Diseases by Inhibiting Bruton’s Tyrosine Kinase
Source: Front Immunol. 2022 Jul 8;13:893899. doi: 10.3389/fimmu.2022.893899 (PMC9304663; doi:10.3389/fimmu.2022.893899)
Supplement: Supplementary file 1 [file DataSheet_1.pdf]

Supplementary information

Material and Methods

Determination of hydroxyproline content in the skin and lung tissue.

Since hydroxyproline originated from collagen, the measurement of hydroxyproline levels can be used as an indicator of collagen content. Skin and lung tissue were homogenized in saline and hydrolyzed in 37% HCL at 120°C for 3 hours. Hydroxyproline concentration was determined by the reaction of oxidized hydroxyproline with 4-(Dimethylamino) benzaldehyde (DMAB), which results in a colorimetric (560nm) product, proportional to the hydroxyproline content. Results were compared against a hydroxyproline standard curve. Values were expressed as mg of hydroxyproline per mg of tissue.

Total IgG and IgM assays

Total serum IgM and IgG levels were measured using commercially available ELISA kits (Cat. # 88-50470 and 88-50400, eBioscience, Inc., CA, USA) according to the manufacturer's manual. All serum samples were diluted 1:500,000 for the IgM assay and 1: 0,000 for the IgG assay. Optical density at 450nm wavelength was measured by microplate reader ELX808 from BioTek Instruments (Winooski, VT) and Ig concentrations were then calculated according to the standard curve.

Serum Autoantibody assays

Serum autoantibodies levels were measured by sandwich ELISA as described in detail previously[17]. Briefly, methylated bovine serum albumin (BSA) was pre-coated onto Immulon 2HB plates before addition of double-stranded DNA, histone, or double stranded DNA and then histones (chromatin). After overnight blocking, mice sera were diluted 1:100 and added to the wells in duplicate. Bound IgG or IgM were detected using alkaline phosphatase-conjugated goat anti-mouse IgG or IgM antibodies (Cat. # 1030-04, and 1021-04, SouthernBiotech, AL, USA) using pNPP (Cat. # N1891, Sigma-Aldrich, MO, USA) as a substrate. The optical density (OD) at 405nm wavelength was measured using a microplate reader ELX808 (BioTek Instruments, VT, USA) and autoantibodies titers were expressed as OD values.

Flow cytometry

Spleens and kidneys were harvested after sacrifice. One third of the spleen in each mouse was used for flow cytometry assay. A single-cell suspension was obtained by crushing the spleen through a 70μm cell strainer, followed by lysis of red blood cells using ACK lysing buffer (Cat. # A1049201, Thermo Fisher Scientific Inc, MA, USA). One kidney per mouse was harvested for flow cytometry analysis. Kidneys were minced with a razor blade and then digested with 1mg/ml collagenase type 4 (Cat. # LS004188, Worthington Biochemical Corp, NJ, USA) for 30 minutes at 37°C in a shaking incubator. After incubation, cells were immediately moved onto ice and the digestion was stopped by adding RPMI media 1640 (Cat. # 12633, Thermo Fisher Scientific Inc, MA, USA). Red blood cells were lysed as described above. Cell clumps were disrupted using a syringe with 22G needle and then forced through a 70μm filter. For flow cytometric analysis, cells were stained with antibodies against B220, CD3, CD4, CD8, CD11b, CD11c, CD21, CD23, CD25, CD45, CD69, CD80, CD86, F4/80, Gr1 (BD Biosciences, CA, USA). Approximately 20,000 events for spleen and 50,000 events for kidney were acquired from each sample. All samples were run on a NovoCyte flow cytometer (ACEA Biosciences, CA, USA). Analysis was performed using Novoexpress (ACEA Biosciences, CA, USA).

Histology evaluation and scoring criteria

Kidney tissue section was subjected to hematoxylin and eosin (H&E) and periodic-acid Schiff (PAS) staining. At least 100 glomeruli were examined per section for evidence of glomerulonephritis and/or tubulointerstitial injury. Glomerulonephritis (GN) score was graded on a 0–4 scale as follows: 0, normal; 1, mild increase in mesangial cellularity and matrix; 2, moderate increase in mesangial cellularity and matrix with thickening of the GBM; 3, focal endocapillary hypercellularity with obliteration of capillary lumina and a substantial increase in the thickness and irregularity of the GBM; and 4, diffuse endocapillary hypercellularity, segmental necrosis, crescents, and hyalinized end-stage glomeruli. Tubulointerstitial injury (TI) score was graded on a 0–4 scale: 0, no lesions; 1, very mild focal dilation and/or very few foci of tubular atrophy; 2, larger number of dilated tubules with widening of interstitium and/or larger number of foci of tubular atrophy; 3, extensive dilation of tubules with cyst formation and widening of interstitium and/or a large number of foci of tubular atrophy; and 4, atrophy of tubules.

For skin tissue, both H&E and Masson staining was performed for each section. The skin histology evaluation includes epidermis, dermis Inflammation, skin fibrosis, as well as vasculitis. Epidermis score

was graded on a 0-1 scale: as 0, intact; 1, Erosion/Ulceration. The dermis Inflammation and skin fibrosis score was both graded on a 0-3 scale: 0, none, 1, mild; 2, moderate; 3, severe. The vasculitis was assessed as 0, none; 1 present. The sum of each score indicated the severity of the skin histology.

The Lung Histopathology Grading included the inflammatory infiltration, airspace dilation, interstitial fibrosis, and vasculitis. Each score was graded on a 0-3 scale, with 0, no pathologic abnormality; 1,mild; 2, moderate; and 3, severe. The sum of each score indicates the severity of change, with a maximum score at 12.

Results

1. BTK Inhibition improved renal function and histopathological changes in mice with spontaneous lupus nephritis:

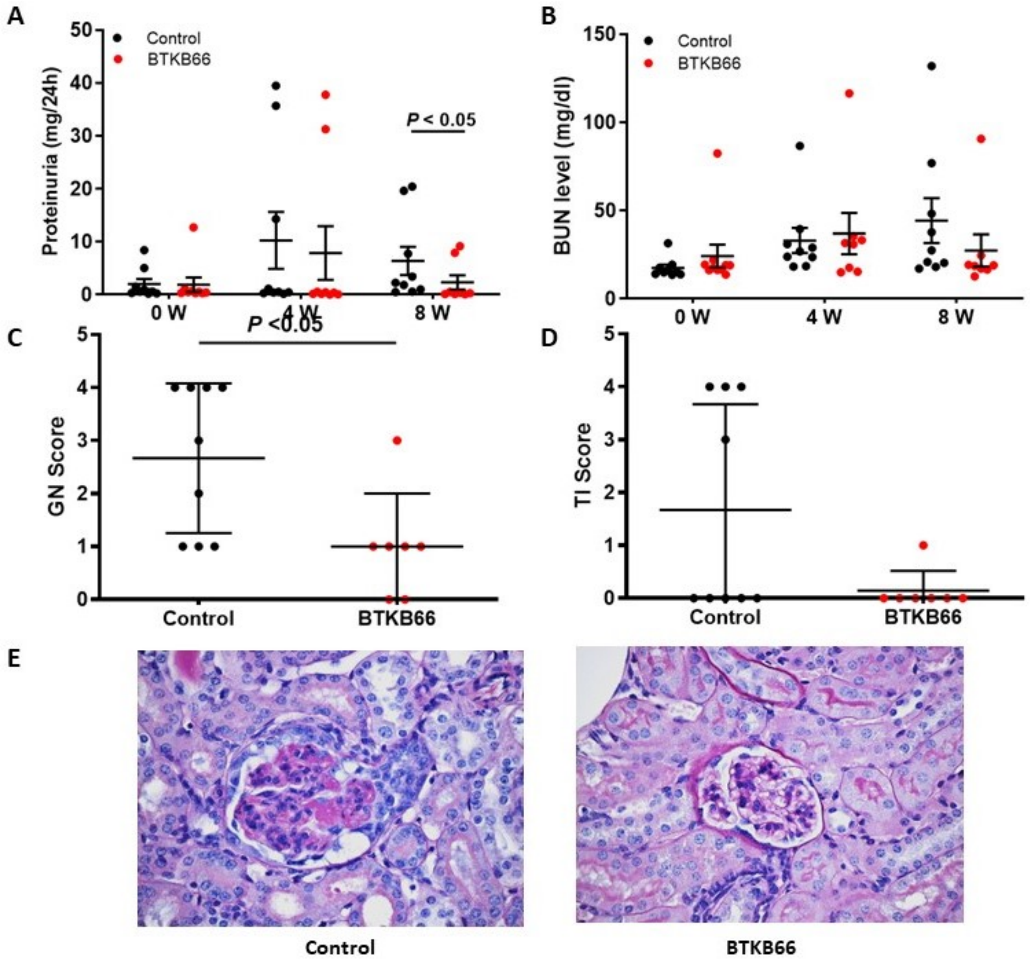

Supplementary Figure S1 BTK Inhibition improved renal function and histopathological changes in mice with spontaneous lupus nephritis: 26-week old BWF1, lupus-prone mice, were treated with BTKB66 for a duration of 8 weeks. (A) Dot plots displaying the change of 24-hour urine protein levels at week 0, 4, and 8 after treatment. (B) Dot plots displaying the change in serum BUN levels at week 0, 4, and 8 after treatment. BUN: blood urea nitrogen. *, *P* <0.05. Renal pathology score was graded by a blinded pathologist. At least 100 glomeruli were examined per section. (C) Blockade of BTK using BTKB66 significantly reduced GN score in 26-week-old BWF1 mice treated for 8 weeks. (D) BTKB66 treatment did not significantly decrease TI score, although a trend towards reduction was noted. (E): Representative image from control and BTKB66 treatment group. GN: glomerulonephritis; TI: tubulointerstitial injury. * *P* <0.05 (non-parametric Mann Whitney U test.

2. BTK inhibitor treatment reduced splenomegaly and regulate serum autoimmunity profile in NZBW/F1

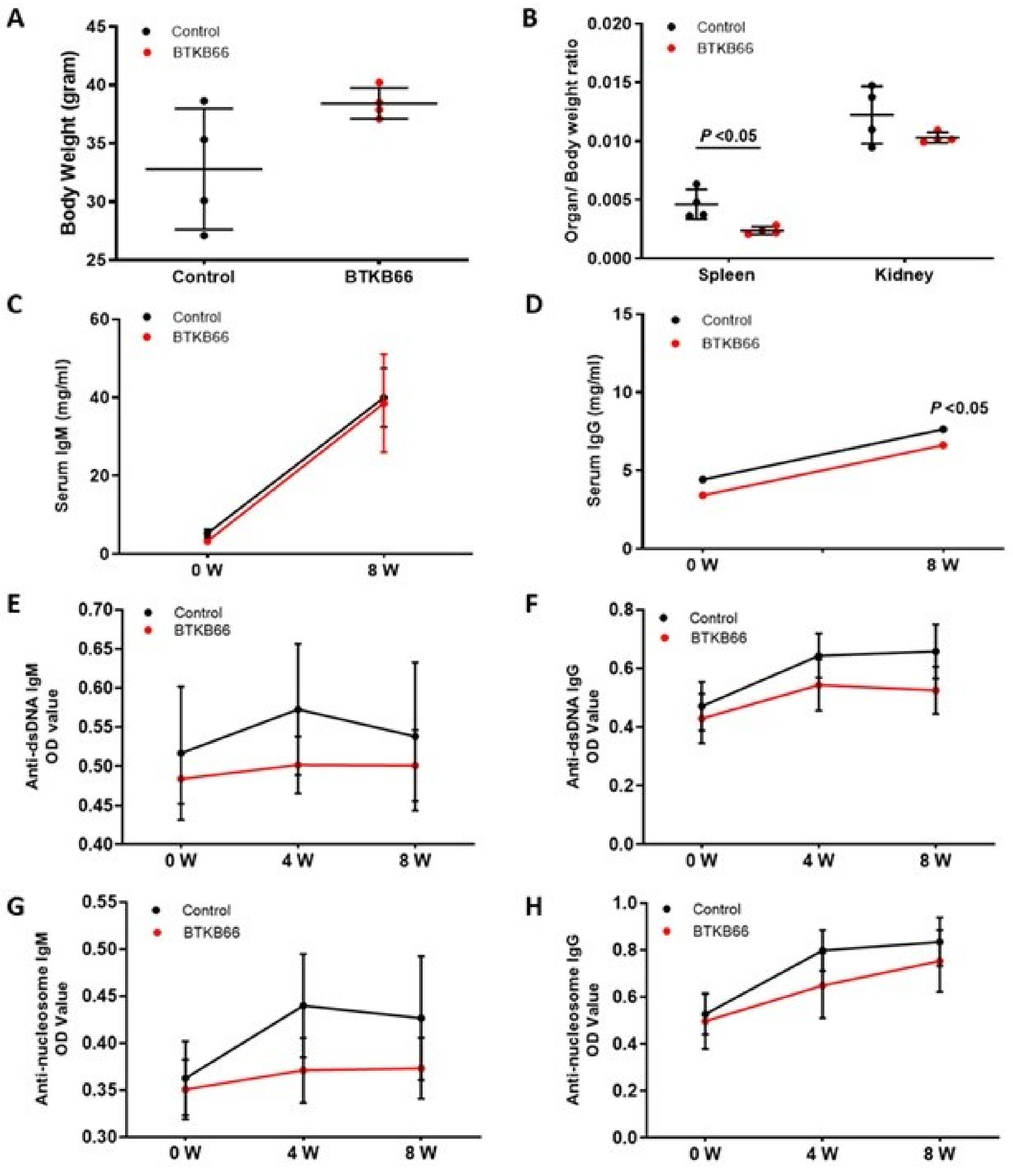

Supplementary Figure S2. The impact of BTK inhibition on splenomegaly and serum autoimmunity profile in NZB/W F1 mice. After 8-week treatment, BTKB66 had no effects on the body weight of mice. (B) BTKB66 treatment significantly reduced splenomegaly in BWF1 mice treated for 8 weeks, as measured using spleen weight normalized by body weight. * P <0.05. Serum levels of total IgG/M and autoantibodies were assayed using ELISA. Plots indicate serum levels of total IgM (A), total IgG (B), anti-dsDNA IgM (C), anti-

dsDNA IgG (D), anti-nucleosome IgM (E), and anti-nucleosome IgG (F) at week 0, week 4, and week 8 after treatment of 26-weeks old BWF1 mice. Grey dotted lines indicate control group and black solid lines indicate treatment group.

3. BTK inhibition improved renal damage in anti-GBM model.

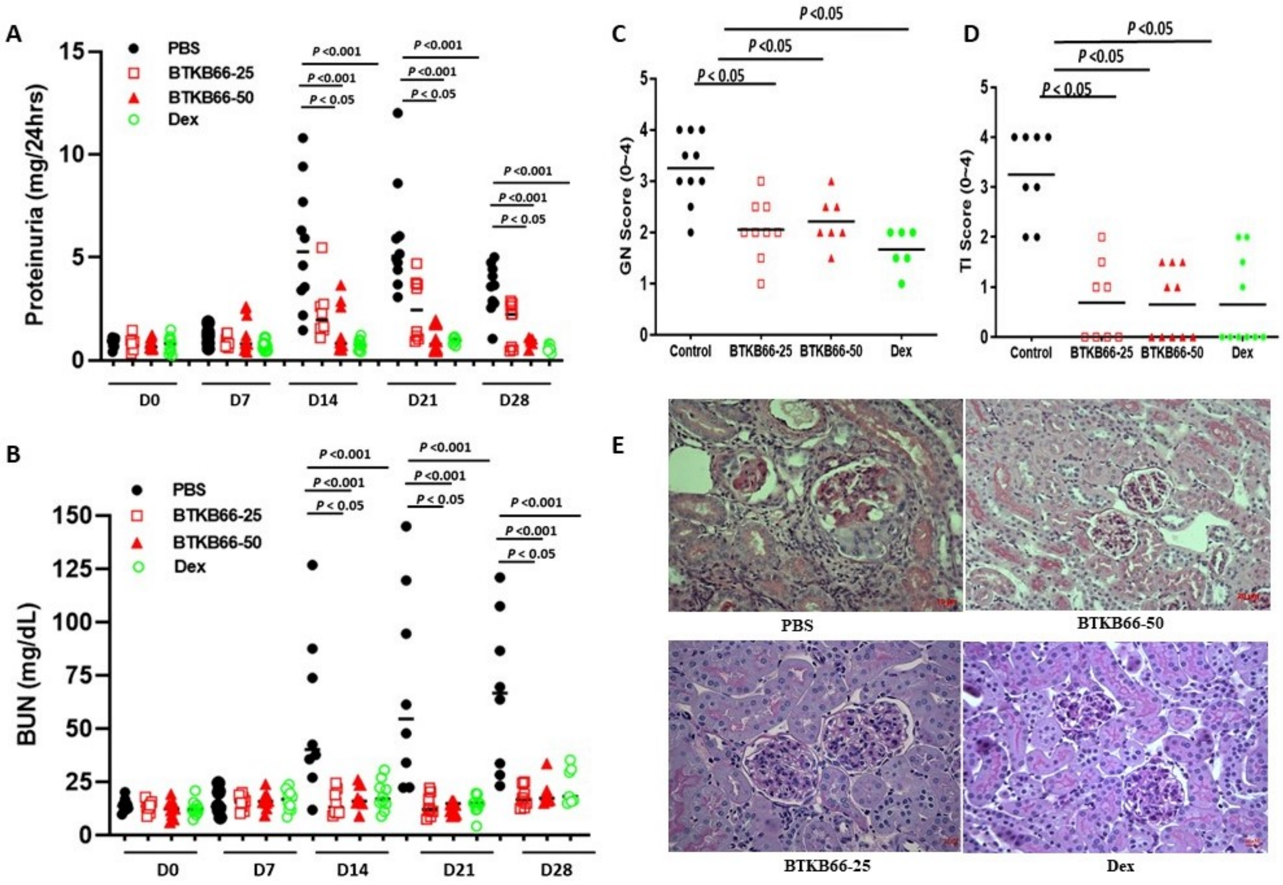

Supplementary Figure S3. The therapeutic efficacy of BTK inhibition on anti-GBM nephritis disease
(A) 24hrs Proteinuria (PU), (B) serum BUN, (C) GN score, (D) TI score, and (E): Representative image from control and BTKB66 treatment group. All groups had similar renal function at baseline (D0 and D7), as indicated by the similar levels of BUN and 24hr PU. However, after anti-GBM antibody challenge, the PBS control group showed worse renal function, with significantly higher BUN and 24hrs PU on D14, D21 as well as D28. Compared to the PBS-treated control group, both the BTKB66 low dose and high dose treatment groups displayed significantly lower levels of BUN and 24hrs PU, accompanied by improved renal pathology changes (P <0.05, One-way ANOVA).

4. BTK inhibition of 26-week-old BWF1 lupus mice significantly reduced peripheral T-cell and myeloid cell activation

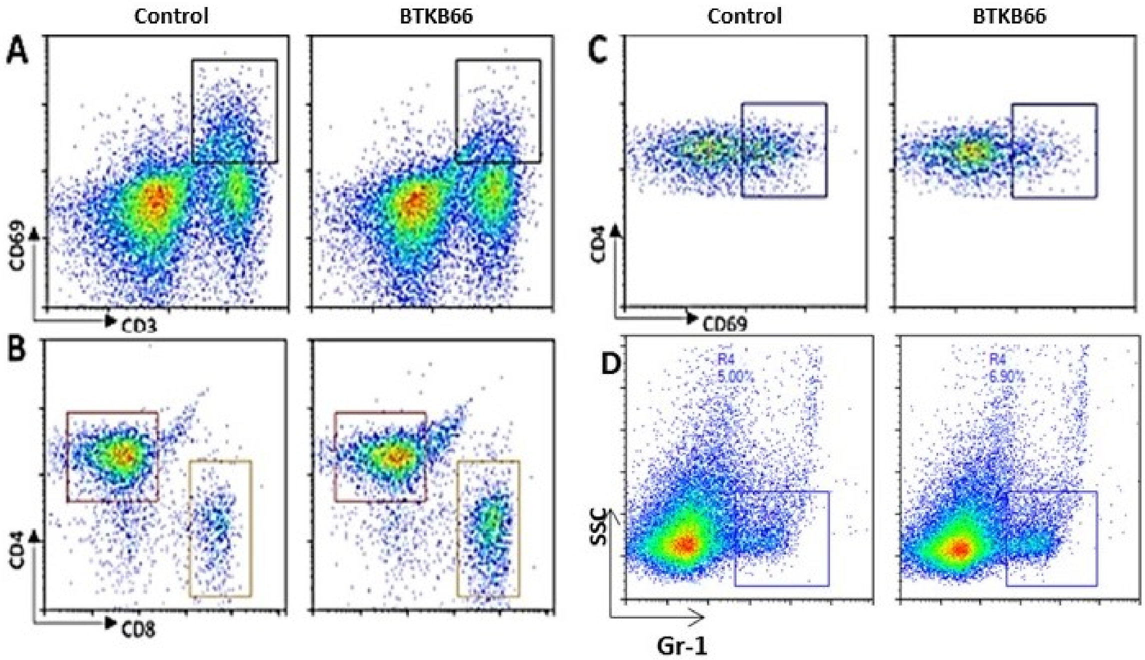

Supplementary Figure S4. Reduced peripheral T-cell and myeloid cell activation after BTK inhibition. (A) The percentage of activated total T cells in the indicated gate. (B) The percentage of CD4+ T cells and CD8+ T cells in the indicated gate. (C) The percentage of activated CD4+ T cells in the indicated gate. (D) The percentage of granulocytes in the indicated gate.

Supplementary Table 1S. Analysis of surface phenotype of intra-renal immune cells by flow cytometry in NZBW F1 mice after 8-week of BTK inhibition.

	% of $CD45^+$		Total cell number		P
	Vehicle	BTKB66	Vehicle	BTKB66	
Total $CD45^+$			2.78 $x10^6$	2.23 x 10^6	NS
T cell population					
$CD45^+CD4^+$	18.3%	22.4%	2.41 $x10^4$	$1.12x10^4$	**
$CD45^+CD4^+CD69^+$	44.8%	25.7%	$5.24x10^3$	$1.28x10^3$	*
$CD45^+CD8$	2.70%	0.91%	$2.64x10^4$	$2.75x10^3$	*
$CD45^+CD8^+CD86^+$	19.2%	6.38%	$2.38x10^4$	$9.62x10^3$	NS
Myeloid					
$CD45^+CD11b^+$	61.1%	55.3%	7.82 $x10^4$	$4.08x10^4$	*
$CD45^+CD11b^+CD86^+$	7.44%	3.87%	1.23 $x10^5$	$8.59x10^4$	*
$CD45^+CD11c^+$	16.7%	18.3%	4.18 $x10^4$	$3.08x10^4$	NS
$CD45^+CD11c^+CD86^+$	13.3%	2.30%	1.52 $x10^5$	$4.06x10^3$	**
$CD45^+Gr1^+$	27.6%	22.6%	5.49 $x10^4$	$3.98x10^4$	NS
$CD45^+Gr1^+CD86^+$	7.83%	1.70%	1.09 $x10^4$	$1.92x10^3$	*
$CD45^+F4/80^+$	20.14%	11.4%	3.99 $x10^4$	$1.88x10^4$	*
$CD45^+F4/80^+CD86^+$	24.26%	22.1%	1.07 x 10^4	$4.60x10^3$	**

All data represent the percentage of parent cells or mean± SD (standard deviation). * *P* <0.05; ***P*<0.01 in the comparison of the number of cells between the Vehicle and BTK inhibitor-treated group.

Supplementary Table 2S. BTK inhibition subdue T cell and Myeloid cell activation in splenocytes of NZBW/F1 mice.

	Control (Mean ± SD)	BTKB66 (Mean ± SD)	*P* value
B cells			
B220$^+$	30.8 ± 8.50	26.0 ± 9.70	NS
B220$^+$CD80$^+$	0.99 ± 1.06	0.85 ± 0.45	NS
B220$^+$ CD21^{hi} CD23^{lo}	11.84 ± 8.08	14.2 ± 6.99	NS
B220$^+$ CD21^{hi} CD23^{lo} CD80$^+$	1.09 ± 2.08	1.13 ± 0.78	NS
B220$^+$ CD21^{lo} CD23^{hi}	43.4 ± 8.48	48.0 ± 10.7	NS
B220$^+$ CD21^{lo} CD23^{hi} CD80$^+$	4.63 ± 3.79	4.93 ± 2.08	NS
T cells			
CD3$^+$	27.8 ± 3.47	30.2 ± 1.91	NS
CD3$^+$CD69$^+$	12.3 ± 2.41	7.85 ± 2.13	*
CD3$^+$CD4$^+$	53.1 ± 1.67	46.4 ± 3.55	**
CD3$^+$CD8$^+$	13.5 ± 0.47	18.5 ± 2.98	*
CD3$^+$CD4$^+$CD69$^+$	19.2 ± 3.82	12.1 ± 3.45	*
CD3$^+$CD8$^+$CD69$^+$	2.15 ± 1.21	1.70 ± 0.40	NS
CD3$^+$CD4$^+$CD25$^+$	5.26 ± 0.73	4.53 ± 0.86	NS
Myeloid cells			
CD11b$^+$ F4/80$^+$	4.30 ± 1.41	5.65 ± 0.66	NS
CD11b$^+$ F4/80$^+$ CD86$^+$	44.8 ± 4.27	51.6 ± 4.67	NS
Gr1$^+$	7.57 ± 0.75	8.98 ± 0.32	*

Gr1$^+$ CD86$^+$	11.3 ± 1.23	12.7 ± 0.86	NS
CD11c$^+$	12.4 ± 2.37	10.0 ± 2.30	NS
CD11c$^+$ CD86$^+$	65.5 ± 4.23	68.7 ± 0.96	NS

All data represent the percentage of parent cells. SD: standard deviation. * P <0.05; ** P <0.01
